# DGM-CM6: A New Model to Predict Distant Recurrence Risk in Operable Endocrine-Responsive Breast Cancer

**DOI:** 10.3389/fonc.2020.00783

**Published:** 2020-05-25

**Authors:** Lei Lei, Xiao-Jia Wang, Yin-Yuan Mo, Skye Hung-Chun Cheng, Yunyun Zhou

**Affiliations:** ^1^Department of Breast Medical Oncology, Chinese Academy of Sciences University Cancer Hospital (Zhejiang Cancer Hospital), Hangzhou, China; ^2^Department of Pharmacology and Toxicology, University of Mississippi Medical Center, Jackson, MS, United States; ^3^Department of Radiation Oncology, Koo Foundation Sun Yat-Sen Cancer Center, Taipei, Taiwan; ^4^Department of Data Science, University of Mississippi Medical Center, Jackson, MS, United States; ^5^Department of Pathology and Laboratory Medicine, Perelman School of Medicine, University of Pennsylvania, Philadelphia, PA, United States; ^6^Raymond G. Perelman Center for Cellular and Molecular Therapeutics, Children's Hospital of Philadelphia, Philadelphia, PA, United States

**Keywords:** clinical-genomic model, breast cancer, distant recurrence, prognosis, endocrine-responsive

## Abstract

To investigate the prognostic value of DGM-CM6 (Distant Genetic Model-Clinical variable Model 6) for endocrine-responsive breast cancer (ERBC) patients, we analyzed 752 operable breast cancer patients treated in a Taiwan cancer center from 2005 to 2014. Among them, 490 ERBC patients (identified by the PAM50 or immunohistochemistry method) were classified by DGM-CM6 into low- and high-risk groups (cutoff <33 and ≥33, respectively). Significant differences were observed between the DGM-CM6 low- and high-risk groups for 10-year distant recurrence-free survival (DRFS) in both lymph node (LN)- (*P* < 0.05) and LN+ patients (*P* < 0.05). Multivariate analysis confirmed the independent strength of DGM-CM6 for the prediction of high- vs. low- risk groups for DRFS (*P* < 0.0001, HR: 6.76, 95% CI, 1.8–25.42) and overall survival (*P* = 0.01, HR: 6.06, 95% CI:1.55–23.47), respectively. In summary, DGM-CM6 may be used to classify low- and high-risk groups for 10-year distant recurrence in both LN- and LN+ ERBC patients in the Asian population. A large scale clinical trial is warranted.

## Highlights

- DGM-CM6 model is capable of predicting long-term distant recurrence risk in both node-negative and positive endocrine responsive breast cancer (ERBC) patients.
- Low-risk ERBC patients identified by DGM-CM6 panel may benefit more from endocrine therapy since the risk of distant relapse at 10 years was <5%.
- High-risk node-positive patients identified by our model had received adjuvant chemotherapy already, and thus receiving prolonged endocrine treatment or adding other agents such as CDK 4/6 inhibitors could be considered in a novel clinical trial as these patients had a 10-year accumulated distant relapse risk of about 20%.
- No prognostic difference was observed between Luminal B and Luminal A in node-negative ERBC patients in our cohort. One of the potential explanations could be that the PAM50 panel might not be optimal for the Asian population.

## Introduction

Although endocrine-responsive breast cancer patients (ERBC) generally have a better outcome than human epidermal growth factor receptor-2 (HER2)-enriched and triple negative breast cancer patients (TNBC), the risk of long-term disease recurrence is unpredictable (1). To maximize the treatment effects, adjuvant chemotherapy has been recommended for high-risk ERCB patients (2). However, it is a challenge to clearly separate high- and low- risk groups for distance recurrence (DR) within the ERCB population, due to the overall better outcomes compared to other subtypes. Therefore, it is critically important to develop models that can accurately predict the group of patients who will benefit from endocrine therapy or chemotherapy, so that all patients can be administered appropriate treatment.

Molecular biomarkers have been very helpful for predicting recurrence-free survival and overall survival in breast cancer patients. Several commercial multi-gene assays have been successfully applied in clinical practice, including 21-gene (Oncotype Dx) recurrence score [RS, (3)], MammaPrint (4), EndoPredict 12 gene (5), PAM50 risk-of-recurrence [ROR, (6)]. However, the performance of these panels has not been found to be optimal in predicting the risk of distant recurrence in node-positive ERBC patients (7). Therefore, the treatment remains ambiguous, especially when these patients have axillary lymph node (LN) metastasis (8–10).

Although the MINDACT trial claimed that MammaPrint could predict node-positive patients who could forgo adjuvant chemotherapy, only 21% of node-positive patients were enrolled in the trial and 52% of them had low genomic risk. Therefore, the results of this trial should be cautiously interpreted considering the small proportion of node-positive patients in the entire study population and a potentially low benefit of 1.5% DR-free survival improvement (11, 12). PAM50 molecular subtypes are closely associated with LN metastasis; however, almost all node-positive patients were classified as high risk (13). Although Luminal A and B are both ERBC, significant differences in clinical outcomes and chemotherapy sensitivity have been reported in several studies (14, 15). Also, Kim et al. reported that the subtype discordant rate between Immunohistochemistry (IHC) and PAM50-based classification was almost 40% (16).

Considering the above limitations, the currently available models for the prediction of long-term DR risk are unsatisfactory in operable ERBC patients, including those who received adjuvant chemotherapy. Therefore, prognostic biomarkers identified from the integration of molecular biomarkers and clinical variables might be more accurate to predict recurrence. Here, we present a previously developed prognostic panel in an Asian cohort study to validate the predictive value for 10-year distant recurrence-free survival (DRFS) in ERBC patients.

## Patients and Methods

### Study Design and Data Description

**Figure 1:**
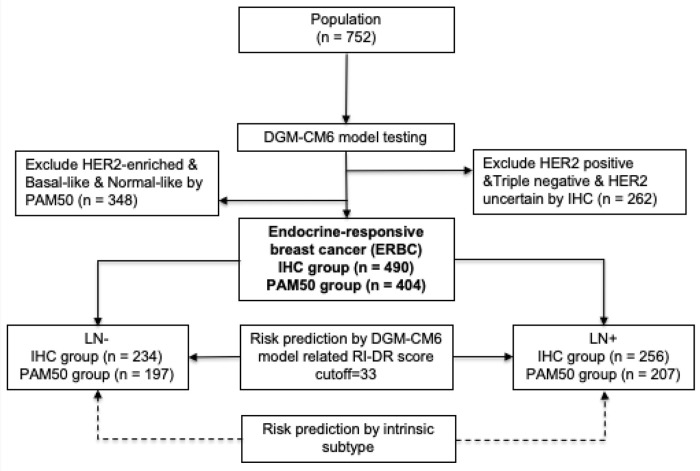
Study design. LN, lymph node; RI-DR, recurrence index for distant recurrence; DGM-CM6, distant genetic model-clinical variable model 6.

The study design is shown in Figure 1. A cohort of 752 breast cancer patients, treated at a free-standing Cancer Center in Taiwan from 2005 to 2014, was included in our retrospective study. The included patients were pN0-2 breast cancer patients who had undergone primary surgery in the form of either mastectomy or breast conserving surgery (BCS). Patients who had pre-operative chemotherapy and cN3, cT4, and/or cM1 disease were excluded. The primary study endpoint was 10-year DRFS, which was defined as the interval from breast cancer surgery until the development of distant recurrence (DR) or death from any cause (17). We defined DR as the spread of breast cancer to any part of the body apart from local and/or regional recurrence. Secondary endpoint was overall survival (OS). The protocol and informed consent documents were reviewed and approved by the institutional review board (IRB) of the hospital (IRB no. 20131001A). The baseline characteristics of the 752 patients are listed in Table S1.

### Identification of ERBC Patients by IHC Staining and Microarray Profiling

ERBC patients were identified by both IHC and microarray profiling from fresh-frozen primary tumor samples. HER2 receptor and/or hormone receptor status was evaluated according to guidelines (18). Patients with ER/PR+, HER2-, grade 1–2 tumors were grouped together as IHC Luminal A subtype; while patients with ER/PR+, HER2-, grade 3 tumors were grouped as IHC Luminal B; and ER/PR+, HER2+ were grouped as Luminal-HER2 (19, 20). The details of the RNA extraction process used for microarray profiling in our study have been previously reported (21). Specifically, raw CEL files from Affymetrix U133 Plus 2.0 platform were pre-processed using the robust multi-array average method in the *affy* package of R software (22). Quantile normalization was performed to reduce potential systematic biases. Each patient was assigned to an intrinsic molecular subtype of breast cancer (Luminal A, Luminal B, HER2-enriched, Basal-like, and Normal-like) by PAM50 method using the *genefu* package of R software (23, 24). The pool of Luminal A/B patients from both IHC (*n* = 490) and PAM50 method (*n* = 404) was defined as ERBC patients (*n* = 499) for down streaming analysis (Table S2).

### Statistical Analyses

The detailed procedure of developing the DGM-CM6 model from the training set (*n* = 112) and testing set (*n* = 46) has been published in our previous study (25). The recurrence index for distant recurrence (RI-DR) score for each patient was computed by the DGM-CM6 model. Patients with DGM-CM6 (RI-DR) scores ≥33 and <33 were defined as high- and low- risk groups for DR, respectively (25).

Wilcoxon rank sum test was used to evaluate the association between DGM-CM6 score vs. IHC- and PAM50 defined Luminal A/B groups. Chi-square test was used to test the association between the risk groups and clinical categorical variables. Kaplan-Meier survival analysis and the log-rank test were used to compare the differences in DRFS and OS between high- and low- risk patients. These survival comparisons were stratified by LN negative (LN-) and positive (LN+) status, respectively. Multivariate Cox regression was used to determine the hazard ratio (HR) for DRFS and OS according to the risk groups adjusted by clinical confounders including age, LN, tumor stages, tumor grade, molecular subtype, and treatment. All statistical analyses were performed using R v.3.4.1.

## Results

### Clinicopathologic Characteristics in ERBC Patients by IHC and PAM50 Classifications

Among the total 499 ERBC patients, 239 were LN- and 260 were LN+. The detailed clinicopathologic characteristics of the patients grouped by LN status are shown in Table 1. According to IHC analyses, 49.9% (249) of subjects were ER/PR+, HER2-, and tumor grade 1–2, 17.8% (89) were ER/PR+, HER2-, and tumor grade 3; and 30.5% (152) were ER/PR+ and HER2+. All patients received treatment and care in accordance with contemporary, evidence-based medicine guided hospital practice guidelines, which are similar to the National Comprehensive Cancer Network guidelines. LN+ patients received more aggressive treatment than LN- patients, including chemotherapy, endocrine therapy, modified radical mastectomy, and adjuvant Trastuzumab treatment. Adjuvant chemotherapy was administered to 87.0% (434) of the patients and adjuvant endocrine therapy to 94.0% (469). Post-mastectomy radiotherapy (PMRT) or regional nodal irradiation (RNI) for BCS patients was administrated in 75.4% (376) of the patients. Among 152 HER2-positive patients, 38.2% (58) received adjuvant Trastuzumab. The characteristics of the patients were well-balanced regardless of the LN status, based on IHC (*p* = 0.358) and PAM50 (*p* = 0.287) subtype classification analyses. Patients with LN positive had significantly poorer (*p* < 0.0001) pathological features, including T stage, lymphovascular invasion (LVI), and grade I/II. The median follow-up time for distant recurrence was 90.6 and 87.5 months for patients with and without LN metastasis, respectively.

**Table 1:** Baseline characteristics of 499 patients with endocrine-responsive breast cancer.

**Variables**	***N***	**LN- (*N* = 239)**	**LN+ (*N* = 260)**	***P*-value**
^*^DGM-CM6 risk group				**0.000**
Low-risk	221	142 (59.4)	79 (30.4)	
High-risk	278	97 (40.6)	181 (69.6)	
**Age**				0.684
>50	201	99 (41.4%)	102 (39.2%)	
≤ 50	298	140 (58.6%)	158 (60.8%)	
**T stage**				**0.000**
T1	234	140 (58.6%)	94 (36.2%)	
T2	253	97 (40.6%)	156 (60%)	
T3	12	2 (0.8%)	10 (3.8%)	
**LVI**				**0.000**
Absent/focal	376	216 (90.4%)	160 (61.5%)	
Prominent	123	23 (9.6%)	100 (38.5%)	
**Tumor Gr**				**0.001**
Gr 1	81	54 (22.6%)	27 (10.4%)	
Gr 2	213	94 (39.3%)	119 (45.8%)	
Gr 3	205	91 (38.1%)	114 (43.8%)	
**Surgery**				**0.000**
BCS	185	120 (50.2%)	65 (25%)	
MRM	314	119 (49.8%)	195 (75%)	
**PMRT or RNI**				**0.000**
No	123	85 (35.6%)	38 (14.6%)	
Yes	376	154 (64.4%)	222 (85.4%)	
**Adjuvant chemotherapy**				**0.000**
No	65	58 (24.3%)	7 (2.7%)	
Yes	434	181 (75.7%)	253 (97.3%)	
**Adjuvant E/T**				0.066
No	30	9 (3.8%)	21 (8.1%)	
Yes	469	230 (96.2%)	239 (91.9%)	
**Adjuvant Trastuzumab**				**0.002**
No	441	223 (93.3%)	218 (83.8%)	
Yes	58	16 (6.7%)	42 (16.2%)	
**IHC subtype**				0.358
ER/PR+ HER2-, Gr 1–2	249	127 (53.1%)	122 (47.3%)	
ER/PR+ HER2-, Gr 3	89	38 (15.9%)	51 (19.8%)	
ER/PR+ HER2+	152	69 (28.9%)	83 (32.2%)	
ER/PR- HER2+	5	4 (1.7%)	1 (0.4%)	
ER/PR- HER2-	2	1 (0.4%)	1 (0.4%)	
**PAM50 subtype**				0.287
Luminal A	192	98 (41%)	94 (36.2%)	
Luminal B	212	99 (41.4%)	113 (43.5%)	
HER2-enriched	40	16 (6.7%)	24 (9.2%)	
Basal-like	15	10 (4.2%)	5 (1.9%)	
Normal-like	40	16 (6.7%)	24 (9.2%)	

DGM-CM6, distant genetic model-clinical variable model 6; LVI, lymphovascular invasion; BCS, breast-conserving surgery; MRM, modified radical mastectomy; PMRT, post-mastectomy radiotherapy; RNI, regional node irradiation; E/T, endocrine therapy; Gr, grade; IHC, immunohistochemistry; ER, estrogen receptor; PR, progesterone Receptor; HER2, Human epidermal growth factor receptor 2.

* *Defined by DGM-CM6: cutoff <33 as low risk, ≥ 33 as high risk*.

### RI-DR Score Is Associated With Lymph Node Status and ERBC Subtypes

**Figure 2:**
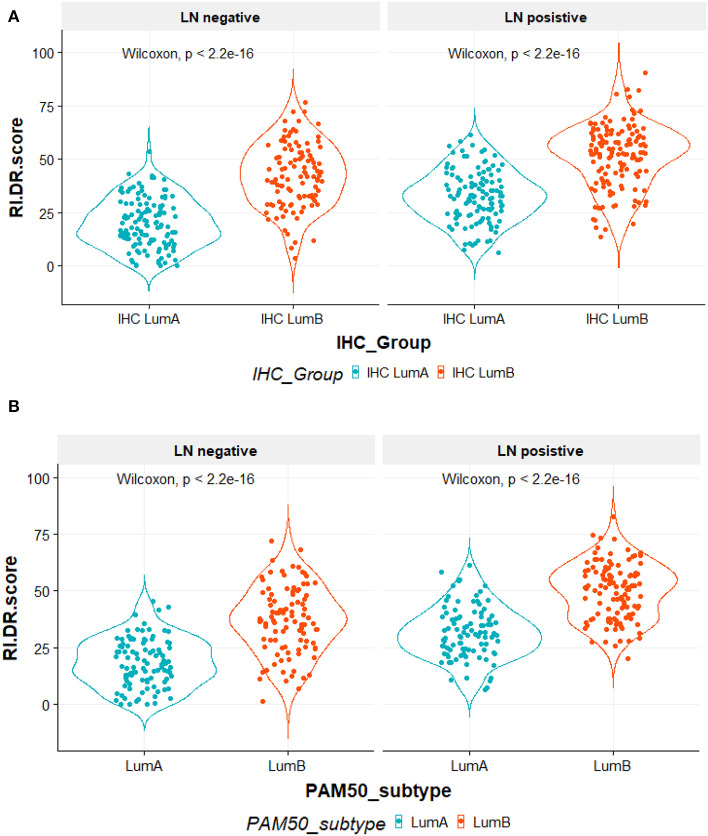
**(A)** All 490 patients were classified according to immunohistochemical (IHC) studies by ER, PR, and HER2 receptors. IHC LumA subtype was defined as patients with ER/PR positive, HER2 negative and grade 1–2 tumors. IHC LumB subtype was defined as patients with ER/PR positive, HER2 negative, and grade 3 tumors. The X-axis is the IHC subtypes, the Y-axis is the RI-DR (recurrence index for distant recurrence) scores. The RI-DR scores in IHC LumA subtype were lower than the scores of IHC LumB. This observation was consistent in both node-negative and -positive patients (Wilcoxon *p* < 2 2e-16). **(B)** All 404 patients were identified by research-based PAM50 intrinsic subtypes using R *genefu* package. Luminal subtypes (LumA and LumB) were classified accordingly. The X-axis is PAM50 subtypes, the Y-axis is RI-DR (recurrence index for distant recurrence) scores. The RI-DR scores of Luminal A were lower than the scores of Luminal B. This observation is consistent in both node-negative and -positive patients (Wilcoxon *p* < 2 2e-16).

RI-DR score is associated with LN status and Luminal A/B subtypes. As shown in Figure 2A, patients were divided by IHC into Luminal A and Luminal B. IHC Luminal B subtype patients had a higher score than patients with Luminal A subtype regardless of LN status (Wilcoxon test, *P* <2.2e-16). Overall, patients with LN metastasis also had higher RI-DR scores than patients without LN metastasis. A similar trend was observed if patients were classified into PAM50-based Luminal A and Luminal B (Figure 2B).

### Prognostic Comparison Between RI-DR Score Defined Risk Group for DR and OS

The cumulative incidence of survival differences between RI-DR defined high- (≥33) and low- (<33) risk groups is shown in b. It can be observed that patients from the high-risk group exhibited significantly poorer prognosis regardless of LN status in the IHC defined ERBC population. Multivariate Cox regression analysis further confirmed that RI-DR score could independently predict high- and low- risk groups for DRFS and OS in IHC defined ERBC population after adjustment for clinical confounders such as age, LN status, stage, grade, and treatment pattern. As shown in Table 2, the prognosis of high-risk group was found to be significantly worse than the low risk group for DRFS (HR=6.76, 95% CI: 1.8–25.42, *p* = 0.005) and OS (HR=6.06, 95% CI: 1.55–23.74, *p* = 0.01). Also, no association was observed between chemotherapy and risk groups (DRFS *p* = 0.163; OS: *p* = 0.195), which implies that our model can predict DRFS and OS of patients regardless of whether they received chemotherapy or not (Table S3).

**Table 2:** Multivariate cox regression analyses for the prognosis of predicted risk groups after adjustment for other clinical variables in case of distant recurrence (DR, *p* = 0.005) and overall survival (OS, *p* = 0.01), respectively.

**Groups**	**DR**		**OS**	
	**HR [95% CI]**	***P*-value**	**HR [95% CI]**	***P*-value**
Risk				
Low-risk	1 (Reference)	-	1 (Reference)	-
High-risk	6.76 [1.8;25.42]	0.005	6.06 [1.55;23.74]	**0.01**
Age (years)				
>50	1 (Reference)	-	1 (Reference)	-
≤ 50	0.86 [0.51;1.44]	0.563	0.68 [0.38;1.19]	0.175
LN				
Positive	1 (Reference)	-	1 (Reference)	-
Negative	1.64 [0.88;3.05]	0.116	1.6 [0.79;3.22]	0.189
Stage				
I	1 (Reference)	-	1 (Reference)	-
II	1.35 [0.76;2.4]	0.3	1.45 [0.76;2.77]	0.264
III	2.08 [0.59;7.3]	0.253	1.59 [0.34;7.46]	0.557
Grade				
1	1 (Reference)	-	1 (Reference)	-
2	2.14 [0.77;5.97]	0.146	1.81 [0.63;5.25]	0.272
3	1.2 [0.4;3.62]	0.748	1.21 [0.38;3.86]	0.752
PAM50				
Luminal A	1 (Reference)	-	1 (Reference)	-
Normal-like	0.6 [0.13;2.64]	0.496	0.7 [0.16;3.16]	0.645
Luminal B	1.58 [0.84;2.98]	0.159	1.29 [0.65;2.59]	0.469
HER2-enriched	0.81 [0.25;2.68]	0.731	1.01 [0.29;3.47]	0.986
RT				
No	1 (Reference)	-	1 (Reference)	-
Yes	0.88 [0.47;1.66]	0.701	0.71 [0.36;1.41]	0.326
CT				
No	1 (Reference)	-	1 (Reference)	-
Yes	0.36 [0.11;1.12]	0.077	0.3 [0.09;1]	0.05
Interaction: CT Vs. risk	0.36 [0.09;1.51]	0.163	0.36 [0.08;1.68]	0.195

The results show that there is no association between chemotherapy and risk groups (DR: p = 0.163; OS: p = 0.195).

*DR, distant recurrence; OS, overall survival; CT, chemotherapy; RT, radiotherapy*.

We further compared the DR and OS risks of our risk groups with PAM50 Luminal A/B groups (Figure 4). Consistent with the IHC cohort results, our RI-DR score could separate patients into high- and low- DR risk groups in case of both LN negative (*p* < 0.0001) and positive patients (*p* = 0.019). With regards to OS our score could separate patients into high-risk and low-risk groups only in LN negative patients (*p* = 0.0047).

## Discussion

Optimal treatment decisions for patients with nodal involvement remain an important goal yet a significant challenge in ERBC patients (11, 26). Following the current breast cancer guidelines for the systemic treatment of ERBC patients, about 97.3% of LN positive ERBC patients received adjuvant chemotherapy. However, 16.9% developed a distant recurrence within 10 years of the primary surgery in our study. Our goal was to find the low-risk group that could avoid toxic chemotherapy and additionally, the high-risk group that should be considered for more aggressive therapy in order to lower the risk of recurrence. The results presented here confirm the robustness of the DGM-CM6 model for the prediction of long-term distant recurrence risk in both LN negative and LN positive ERBC patients. Multivariate analysis demonstrated that our DGM-CM6 panel could independently predict the prognosis of ERBC patients for both DRFS and OS after adjustment for clinical confounders including molecular subtypes, LN status, and other clinical factors, regardless of whether the patients received chemotherapy or not (Table 2). Of note, our results also highlighted that within the ERBC population, not all ER/PR+ HER2- samples are Luminal-like since basal-like and HER2-enriched samples could also be identified (Table S2). This suggests that our model can successfully predict the DR risk in ERBC patients based on the status of three IHC biomarkers what might be considerably cost-effective.

**Figure 3:**
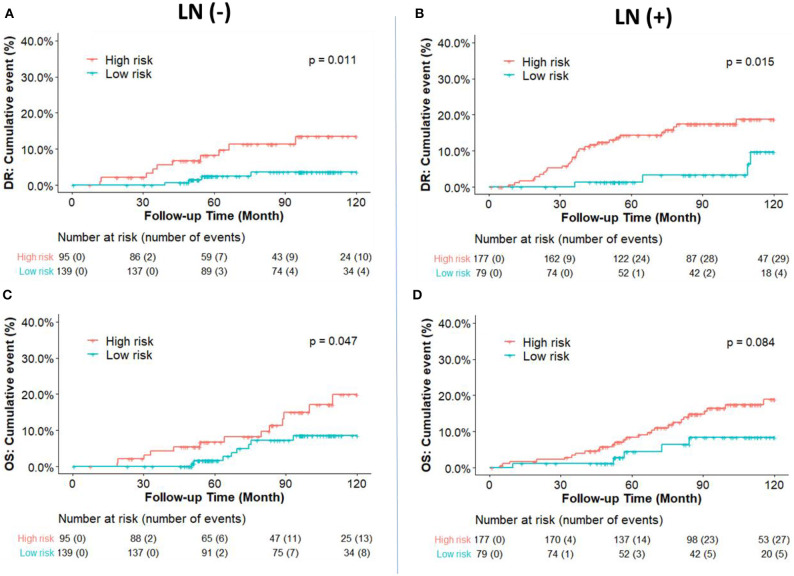
Kaplan-Meier plot survival curves for distant recurrence **(A,B)** and overall survival **(C,D)** in node-negative and node-positive patients according to the IHC classification. RI-DR (recurrence index for distant recurrence) could divide node-negative and positive patients into low- and high-risk groups. *P*-value was calculated by log-rank test.

From a clinical perspective, patients who experience no recurrence after 5 years of endocrine therapy and have a sufficiently low risk should not be recommended an extension of the endocrine therapy. Therefore, we hypothesized that the patients in our study who were predicted by the DGM-CM6 panel to be low risk in the LN- ERBC population may benefit more from endocrine therapy than chemotherapy, since the <5% risk of distant relapse at 10 years implies they may safely avoid adjuvant chemotherapy and prolonged endocrine therapy (current cohort was treated with 5-year endocrine therapy). Whereas, patients with high risk in the LN+ ERBC population had received adjuvant chemotherapy already, and thus received prolonged endocrine treatment and were also enrolled in a novel clinical trial as RI-DR-high-risk patients as they had a 10-year accumulated distant relapse risk of about 20% (Figure 3).

**Figure 4:**
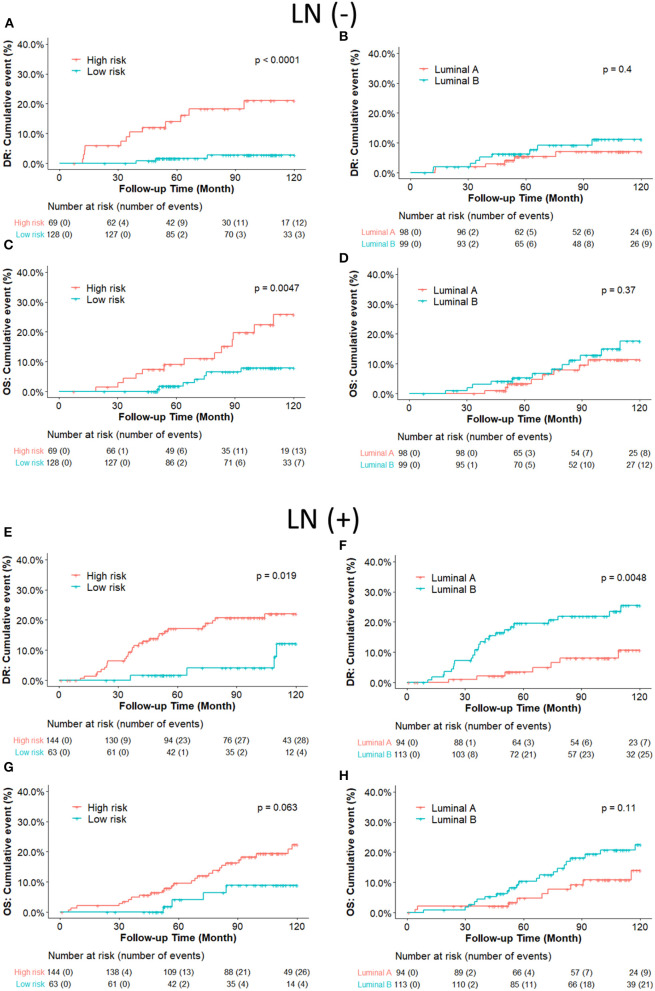
**(A–D)** Kaplan-Meier plot survival curves for distant recurrence and overall survival in node-negative patients according to the PAM50 classification. RI-DR (recurrence index for distant recurrence) could significantly divide patients into low- and high-risk groups **(A,C)**; while Luminal A subtype could not be differentiated well from the Luminal B subtype, which is supposed to have higher risk than Luminal A **(B,D)**. **(E–H)** Kaplan–Meier plot survival curves for distant recurrence and overall survival in node-positive patients according to the PAM50 classification. Both RI-DR risk groups and intrinsic Luminal A/B showed significant prognostic difference in risk of distant recurrence in the node-positive population **(E,F)**, but no significance in overall survival **(G,H)** (31).

Intrinsic subtypes Luminal A and B defined by PAM50 have been well-known to behave differently with respect to clinical outcome and treatment sensitivities (27). Luminal B is more aggressive with more propensity to develop relapse and resistance to endocrine therapy than Luminal A (28, 29). Intrinsic subtypes could provide precise information for recurrence risk prediction in early breast cancer (30). However, we found that there was no prognostic difference between Luminal B and Luminal A in LN-negative ERBC patients in our cohort, while our DGM-CM6 panel performed better in separating high and low-risk groups (Figure 4). One of the potential explanations could be that the PAM50 panel may not be optimal for the Asian population, especially for low-risk ERBC patients. As a result, LN- patients in our study had a good prognosis even if they were classified as luminal B by PAM50. This observation is consistent with those of previous studies that Asian women had significantly reduced relative odds of other PAM50 subtypes vs. Luminal A in the prediction of short and long-term prognostic outcomes (32, 33). Moreover, intrinsic Luminal A and Luminal B subtypes can only be derived from microarray-based data, and thus are commercially expensive. Furthermore, increasing evidence about the discordant results between PAM50 based intrinsic subtypes and IHC based subtypes has been reported (16, 34, 35). Consequently, we validated the predictive risk value of the DGM-CM6 model in both IHC and intrinsic subtype cohorts in order to avoid the discordance issue among different classifiers. The strength of our panel is that it has prognostic value in both IHC- and microarray-based data, thus demonstrating the clinical utility of the DGM-CM6 in a practical setting.

However, some potential limitations of this study need to be noted. Firstly, we included chemo-treated patients in our study, which could possibly lower the recurrence risk in these patients. However, our multivariate Cox regression analysis for DRFS and OS showed that there was no association between chemotherapy and DGM-CM6 risk groups. This result makes sense since previous studies have shown that adjuvant chemotherapy could not stop the development of late recurrence in ERBC patients, especially in HER2-negative tumors (36, 37). Secondly, menopause status should be discussed carefully, but we were unable to obtain well-documented menstruation information in this retrospective study. Thirdly, it would be much more interesting to have the PAM50-based ROR scores as the control panel in our study. However, the PAM50 intrinsic subtype classification could also have more prognostic value than pathological characteristics (38). In this large cohort study, multivariate analysis showed that both of intrinsic subtypes and ROR risk classification yielded strong prognostic information in early-stage breast cancer. The final limitation of our study is that the current cutoff of DGM-CM6 score (≥33) may not be suitable if we attempt to use it for all specific categories of patients (i.e., different subtypes, LN+ numbers). Nevertheless, tumor recurrence and treatment outcome are a product of complex interactions between tumor subtypes, immune system, the status of lymph nodes, tumor-stroma interactions, and do not depend solely on luminal A or luminal B type. Additionally, some immune therapies may play a role in the outcome and recurrence of the disease. Consequently, it is necessary to consider the role of the immune system, especially the non-specific properties and role of natural killer (NK) cells in lymph nodes, which has been found to be significant in previously studies (38–40). Therefore, standardization of a biomarker cutoff applicable for patients of all categories would not be realistic unless all training/testing/validating sets could be unified and well-balanced for all characteristics.

## Conclusions

In summary, this study demonstrated the prognostic value of the DGM-CM6 panel for making treatment decisions in ERBC women, regardless of LN status. Importantly, our panel consistently showed good performance in both IHC- and microarray-derived ERBC candidates, thus solving the discordance issue reported by other studies. Finally, as far as we know, DGM-CM6 is a new edition of the first generation of multi-gene expression predictive model developed for Asian breast cancer patients which combined genome and clinical-pathological information.

## Data Availability Statement

The datasets during and/or analyzed during the current study available from the corresponding author on reasonable request.

## Ethics Statement

The studies involving human participants were reviewed and approved by The protocol and informed consent documents were reviewed and approved by the institutional review board (IRB) of the Koo Foundation Sun Yat-Sen Cancer Center in Taipei, Taiwan (IRB no. 20131001A). The patients/participants provided their written informed consent to participate in this study. Written informed consent was obtained from the individual(s) for the publication of any potentially identifiable images or data included in this article.

## Author Contributions

LL contributed to project design and wrote the manuscript. X-JW and Y-YM contributed to results interpretation. YZ and SC led the project, provided project design guidance and prepared the manuscript. All authors read and approved the manuscript.

## Conflict of Interest

SC is currently applying for the patent relating to the content of this manuscript. The remaining authors declare that the research was conducted in the absence of any commercial or financial relationships that could be construed as a potential conflict of interest.

## Abbreviations

DGM-CM6: distant genetic model-clinical variable model 6
LVI: lymphovascular invasion
BCS: breast-conserving surgery
IHC: immunohistochemistry
ER: estrogen receptor
PR: progesterone Receptor
HER2: Human epidermal growth factor receptor 2.
