# Supplementary material for: DGM-CM6: A New Model to Predict Distant Recurrence Risk in Operable Endocrine-Responsive Breast Cancer
Source: Front Oncol. 2020 May 25;10:783. doi: 10.3389/fonc.2020.00783 (PMC7263173; doi:10.3389/fonc.2020.00783)
Supplement: Supplementary file 1 [file Table_1.docx]

Table S1. Baseline characteristics of subjects in the internal validation dataset (N=752).

| Variables | All patients | Low risk^*^(N = 232) | High risk^*^(N = 520) | *P-*value |
| --- | --- | --- | --- | --- |
| **Median follow-up (Months)** | 95.8(0.6-169.3) | 95.8(0.6-169.3) | 95.8(0.9-164.5) | 0.2525 |
| **Age** |  |  |  | **0.0001** |
| <40 | 121 | 21(9.1%) | 100(19.2%) |  |
| 40-60 | 519 | 163(70.3%) | 356(68.5%) |  |
| >60 | 112 | 48(20.7%) | 64(12.3%) |  |
| **T stage** |  |  |  | **<0.0001** |
| T1 | 327 | 152(65.5%) | 175(33.7%) |  |
| T2 | 408 | 76(32.8%) | 332(63.9%) |  |
| T3 | 17 | 4(1.7%) | 13(2.5%) |  |
| **N stage** |  |  |  | **<0.0001** |
| N0 | 364 | 152(65.5%) | 212(40.8%) |  |
| N1 | 282 | 67(28.9%) | 215(41.4%) |  |
| N2 | 106 | 13(5.6%) | 93(17.9%) |  |
| **ER and PR status** |  |  |  | **<0.0001** |
| Negative(both) | 260 | 14(6.0%) | 246(47.3%) |  |
| ER or PR (+) | 492 | 218(94.0%) | 274(52.7%) |  |
| **HER2 overexpression** |  |  |  | **<0.0001** |
| Negative | 492 | 198(85.3%) | 294(56.5%) |  |
| Positive | 257 | 34(14.7%) | 223(42.9%) |  |
| Uncertain | 3 | 0(0.0%) | 3(0.6%) |  |
| **LVI** |  |  |  | **<0.0001** |
| Absent/focal | 582 | 201(86.6%) | 381(73.3%) |  |
| Prominent | 170 | 31(13.4%) | 139(26.7%) |  |
| **Tumor grade** |  |  |  | **<0.0001** |
| Grade 1 | 83 | 74(31.9%) | 9(1.7%) |  |
| Grade 2 | 238 | 123(53.0%) | 115(22.1%) |  |
| Grade 3 | 431 | 35(15.1%) | 396(76.2%) |  |
| **PMRT or RNI** |  |  |  | **<0.0001** |
| No | 184 | 78(33.6%) | 106(20.4%) |  |
| Yes | 568 | 154(66.4%) | 414(79.6%) |  |
| **Adjuvant C/T** |  |  |  | **<0.0001** |
| No | 82 | 54(23.3%) | 28(5.4%) |  |
| Yes | 670 | 178(76.7%) | 492(94.6%) |  |
| **Adjuvant E/T** |  |  |  | **<0.0001** |
| No | 281 | 20(8.6%) | 261(50.2%) |  |
| Yes | 471 | 212(91.4%) | 259(49.8%) |  |
| **Adjuvant Trastuzumab** |  |  |  | **<0.0001** |
| No | 655 | 220(94.8%) | 435(83.7%) |  |
| Yes | 97 | 12(5.2%) | 85(16.4%) |  |
| IHC subtype |  |  |  | **<0.0001** |
| ER/PR+ HER2-, Grade 1-2 | 249 | 171(73.7%) | 78(15.0%) |  |
| ER/PR+ HER2-, Grade 3 | 89 | 19(8.2%) | 70(13.5%) |  |
| ER/PR+ HER2+ | 152 | 28(12.1%) | 124(23.9%) |  |
| ER-, PR-, HER2+ | 105 | 6(2.6%) | 99(19.0%) |  |
| ER-, PR-, HER2- | 154 | 8(3.5%) | 146(28.1%) |  |

^*^ Defined by DGM-CM6: cutoff < 33 as low risk, $\geq$33 as high risk.

Abbreviations: BCS, breast-conserving surgery; C/T, chemotherapy; E/T, endocrine therapy; LVI, lymphovascular invasion; MRM, modified radical mastectomy; PMRT, post-mastectomy radiotherapy; RNI, regional node irradiation
