# Supplementary material for: DGM-CM6: A New Model to Predict Distant Recurrence Risk in Operable Endocrine-Responsive Breast Cancer
Source: Front Oncol. 2020 May 25;10:783. doi: 10.3389/fonc.2020.00783 (PMC7263173; doi:10.3389/fonc.2020.00783)
Supplement: Supplementary file 2 [file Table_2.docx]

Table S2. Distribution of ERBC patients by PAM50 intrinsic subtypes and IHC subtypes.

|  | Basal | Her2 | LumA | LumB^*^ | Normal |
| --- | --- | --- | --- | --- | --- |
| ER(+), HER2(-),Grade1-2 | 4 | 1 | 148 | 79 | 17 |
| ER(+), HER2(-),Grade3 | 8 | 3 | 19 | 53 | 6 |
| ER(+), HER2(+) | 3 | 36 | 20 | 76 | 17 |
| ER(-), HER2(+) | 15 | 76 | 4 | 1 | 9 |
| ER(-), HER2(-) | 114 | 23 | 1 | 1 | 15 |

^*^Two luminal B patients by PAM50 were not having IHC results.

Abbreviations: ERBC, endocrine-responsive breast cancer; LUMA, luminal A; LUMB, luminal B.
