# Supplementary material for: DGM-CM6: A New Model to Predict Distant Recurrence Risk in Operable Endocrine-Responsive Breast Cancer
Source: Front Oncol. 2020 May 25;10:783. doi: 10.3389/fonc.2020.00783 (PMC7263173; doi:10.3389/fonc.2020.00783)
Supplement: Supplementary file 3 [file Table_3.docx]

Table S3. Multivariate cox regression analyses for the prognosis of ERBC patients who ever received adjuvant chemotherapy (N=434).

| Groups | DR | | OS | |
| --- | --- | --- | --- | --- |
|  | HR [95% CI] | P value | HR [95% CI] | P value |
| Risk |  |  |  |  |
| Low-RISK | 1(Reference) | - | 1(Reference) | - |
| high-risk | 2.79 [1.16;6.66] | **0.021** | 2.83 [1.04;7.66] | **0.041** |
| Age (years) |  |  |  |  |
| >50 | 1(Reference) | - | 1(Reference) | - |
| ≤50 | 1.29 [0.71;2.33] | 0.405 | 1.14 [0.58;2.23] | 0.696 |
| LN |  |  |  |  |
| positive | 1(Reference) | - | 1(Reference) | - |
| Negative | 1.24 [0.66;2.31] | 0.508 | 1.14 [0.56;2.32] | 0.712 |
| stage |  |  |  |  |
| i | 1(Reference) | - | 1(Reference) | - |
| II | 1.22 [0.66;2.26] | 0.525 | 1.29 [0.63;2.62] | 0.489 |
| iii | 1.32 [0.29;5.91] | 0.719 | 0.94 [0.12;7.44] | 0.951 |
| Grade |  |  |  |  |
| 1 | 1(Reference) | - | 1(Reference) | - |
| 2 | 1.43 [0.41;5.02] | 0.577 | 1.18 [0.32;4.31] | 0.799 |
| 3 | 0.74 [0.19;2.89] | 0.667 | 0.74 [0.18;3.01] | 0.669 |
| PAM50 |  |  |  |  |
| Luminal a | 1(Reference) | - | 1(Reference) | - |
| Normal-LIKE | 0.93 [0.21;4.18] | 0.922 | 1.21 [0.26;5.61] | 0.804 |
| LumINAL B | 1.67 [0.8;3.47] | 0.172 | 1.24 [0.54;2.84] | 0.612 |
| Her2-E | 0.95 [0.27;3.28] | 0.933 | 1.07 [0.29;3.92] | 0.918 |
| RT |  |  |  |  |
| no | 1(Reference) | - | 1(Reference) | - |
| Yes | 0.93 [0.45;1.92] | 0.844 | 0.66 [0.31;1.41] | 0.282 |

Abbreviations: ERBC, endocrine-responsive breast cancer; DR, distant recurrence; OS: overall survival
